# Particulate Soup: Identifying the Most Toxic Constituents of PM_2.5_

**DOI:** 10.1289/ehp.118-a130a

**Published:** 2010-03

**Authors:** Rebecca Clay Haynes

**Affiliations:** **Rebecca Clay Haynes** has written for *EHP* since 1993. Her work has also appeared on National Public Radio and in the *Christian Science Monitor* and *The Environmental Forum*. In addition, she is the author of two children’s science books related to astronomy and space exploration

Chronic exposure to air pollution has long been linked with cardiopulmonary-related mortality, and ambient fine particulate matter (PM_2.5_) is often considered a primary cause of this association. Once inhaled, these particles can cause inflammation and oxidative stress. This in turn may result in systemic effects, including the buildup of plaque deposits that can lead to heart attacks and strokes. A new study comparing air pollution exposure with health data gathered over a 5-year span now takes a closer look at which individual constituents of PM_2.5_ may be most likely responsible for associations between ambient air quality and mortality **[*EHP*** **118:363–369; Ostro et al.]**.

Ambient PM_2.5_ contains solid and liquid particles from many sources, particularly from fossil fuel combustion; among other constituents, it contains elemental and organic carbon, sulfates, nitrates, iron, potassium, silicon, and zinc. The National Research Council has highlighted the importance of routinely collecting toxicity data on particle constituents to help refine air quality standards, target control strategies, and enhance the accuracy of health impact assessments.

In the current study, the authors used data from the California Teachers Study for 45,000 active and former female public school professionals. The teachers lived within 8 or 30 km of monitors that collected data on PM_2.5_ between June 2002 and July 2007. The large amount of individual-level data provided by participants allowed researchers to control for risk factors that could possibly confound the analysis. Smoking rates and indoor occupational exposures, for example, were very low in this study cohort, making it easier to identify an independent effect of outdoor air pollution. Air pollution measurements were generally taken twice a week, and information from health questionnaires also helped determine individual exposure.

Of 8 constituents studied, organic carbon and sulfates were found to be most strongly associated with all-cause, cardiopulmonary, ischemic heart disease, and pulmonary mortality. Even modest concentrations of these 2 constituents were associated with mortality from all 4 causes. According to the authors, the study provides new information to help focus and streamline regulatory efforts on a variety of sources of PM_2.5_, including gasoline and diesel fuel, and other combustion activities. They write that the reduction of ambient PM_2.5_, particularly from fuel combustion, may offer significant public health benefits.

## Figures and Tables

**Figure:**
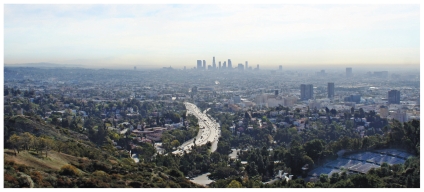
PM_2.5_ contributes to the haze that blankets metropolitan areas.

